# Supplementary material for: The Association Between Fast Food Consumption and Inflammatory Bowel Disease: A Case-Control Study and Meta-Analysis
Source: Nutrients. 2025 May 28;17(11):1838. doi: 10.3390/nu17111838 (PMC12158017; doi:10.3390/nu17111838)
Supplement: Supplementary file 1 [file nutrients-17-01838-s001.zip › nutrients-3649383-supplementary.pdf]

Supplementary Table S1. Quality assessment of the studies included in the meta-analysis using the Newcastle-Ottawa Scale.

Item	Almofarreh	Qalqili	DeClercq	Niewiadomski	Persson
Case definition	*	*	*	*	*
Representativeness of cases	*	*	*	*	*
Selection of controls	--	*	*	*	*
Definition of controls	*	*	*	*	*
Comparability	*	*	*	--	*
Ascertainment of exposure	--	--	--	--	--
The same method of ascertainment for cases and controls	*	*	*	*	*
Nonresponse rate	--	--	--	--	--
Overall score (total number of asterisks)	5	6	6	5	6

Overall score: 0–3 (low quality), 4–6 (medium quality), and 7–9 (high quality).

Supplementary Table S2. GRADE summary.

Domain	Assessment	Justification
Study design	●●○○ (Low)	All studies are observational (case-control or cross-sectional).
Risk of Bias	Serious	All studies used self-reported dietary data, which may introduce recall and selection bias. Most studies did not adjust for important confounders.
Inconsistency	Serious	Exposure definitions and recall periods for fast food consumption varied across studies, limiting comparability.
Indirectness	Not Serious	Populations, exposures, and outcomes are directly applicable to the review question.
Imprecision	Not Serious	ORs and CIs were calculated.
Publication Bias	Not Serious	The funnel plot showed no significant asymmetry.

```
              estimate    se  zval  pval  ci.lb ci.ub      Q    Qp  tau2     I2    H2
-Niewiadomski    0.831 0.546 1.523 0.128 -0.239 1.901 28.620 0.000 1.051 89.518 9.540
-Persson         0.778 0.489 1.591 0.112 -0.180 1.736 28.129 0.000 0.851 89.335 9.376
-Qalqili         1.209 0.337 3.589 0.000  0.548 1.869 11.577 0.009 0.327 74.086 3.859
-DeClercq        0.978 0.521 1.878 0.060 -0.043 1.999 25.701 0.000 0.943 88.327 8.567
-Almofarreh      0.595 0.372 1.597 0.110 -0.135 1.325 11.801 0.008 0.407 74.579 3.934
```

Supplementary Figure S1. The impact of removing studies one by one and combining the remaining studies in a meta-analysis investigating the association between fast food consumption and ulcerative colitis.

```
              estimate    se  zval  pval  ci.lb ci.ub      Q    Qp  tau2     I2     H2
-Niewiadomski    1.022 0.593 1.723 0.085 -0.140 2.184 18.208 0.000 0.922 89.016  9.104
-Persson         0.904 0.480 1.885 0.059 -0.036 1.844 20.988 0.000 0.619 90.471 10.494
-DeClercq        1.282 0.358 3.579 0.000  0.580 1.985  9.458 0.009 0.288 78.853  4.729
-Almofarreh      0.648 0.315 2.058 0.040  0.031 1.266  4.965 0.084 0.176 59.715  2.482
```

Supplementary Figure S2. The impact of removing studies one by one and combining the remaining studies on the meta-analysis investigating the association between fast food consumption and Crohn’s disease.

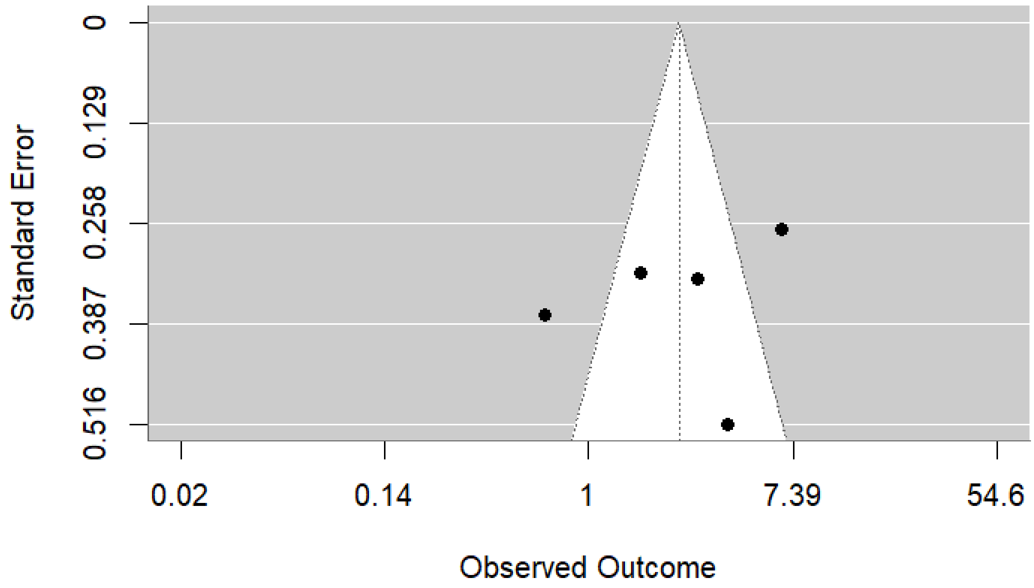

Supplementary Figure S3. Funnel plot of the studies investigating the association between fast food consumption and ulcerative colitis.

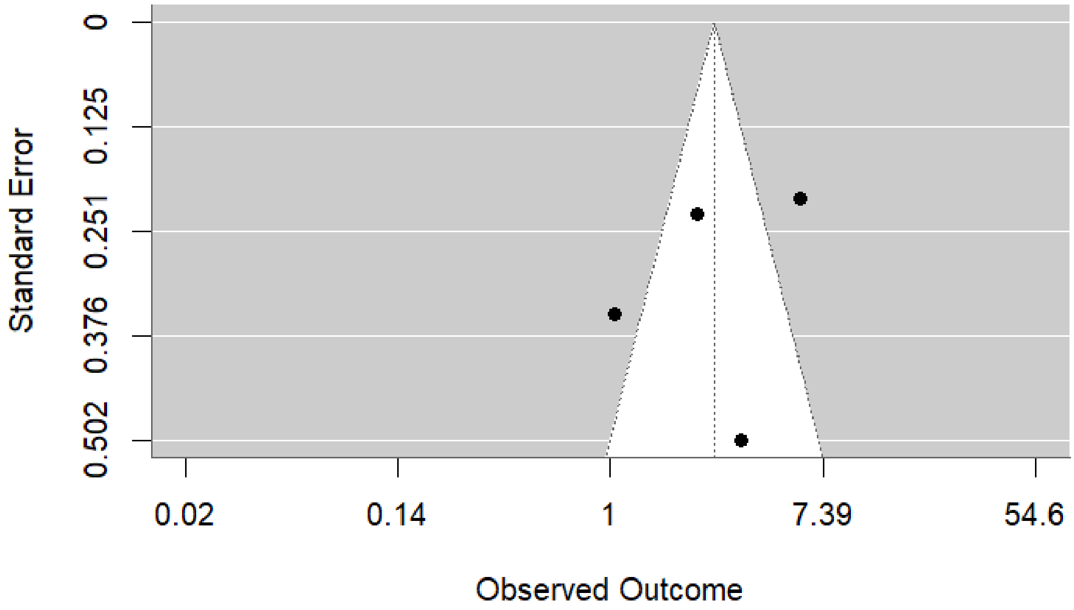

Supplementary Figure S4. Funnel plot of the studies investigating the association between fast food consumption and Crohn's disease.
